# The UDP-glucose: glycoprotein glucosyltransferase (UGGT), a key enzyme in ER quality control, plays a significant role in plant growth as well as biotic and abiotic stress in *Arabidopsis thaliana*

**DOI:** 10.1186/s12870-015-0525-2

**Published:** 2015-05-28

**Authors:** Francisca Blanco-Herrera, Adrián A. Moreno, Rodrigo Tapia, Francisca Reyes, Macarena Araya, Cecilia D’Alessio, Armando Parodi, Ariel Orellana

**Affiliations:** Centro de Biotecnología Vegetal, Facultad de Ciencias Biológicas, Universidad Andrés Bello, Avenida República 217, Santiago, 837-0146 RM Chile; FONDAP Center for Genome Regulation, Santiago, RM Chile; Fundación Instituto Leloir and IIBBA, CONICET, Buenos Aires, Argentina; School of Sciences, University of Buenos Aires, Buenos Aires, Argentina

**Keywords:** UGGT, Endoplasmic reticulum, Abiotic stress, Biotic stress

## Abstract

**Background:**

UDP-glucose: glycoprotein glucosyltransferase (UGGT) is a key player in the quality control mechanism (ER-QC) that newly synthesized glycoproteins undergo in the ER. It has been shown that the UGGT Arabidopsis orthologue is involved in ER-QC; however, its role in plant physiology remains unclear.

**Results:**

Here, we show that two mutant alleles in the At1g71220 locus have none or reduced UGGT activity. In wild type plants, the *AtUGGT* transcript levels increased upon activation of the unfolded protein response (UPR). Interestingly, mutants in *AtUGGT* exhibited an endogenous up–regulation of genes that are UPR targets. In addition, mutants in *AtUGGT* showed a 30 % reduction in the incorporation of UDP-Glucose into the ER suggesting that this enzyme drives the uptake of this substrate for the CNX/CRT cycle. Plants deficient in UGGT exhibited a delayed growth rate of the primary root and rosette as well as an alteration in the number of leaves. These mutants are more sensitive to pathogen attack as well as heat, salt, and UPR-inducing stressors. Additionally, the plants showed impairment in the establishment of systemic acquired resistance (SAR).

**Conclusions:**

These results show that a lack of UGGT activity alters plant vegetative development and impairs the response to several abiotic and biotic stresses. Moreover, our results uncover an unexpected role of UGGT in the incorporation of UDP-Glucose into the ER lumen in *Arabidopsis thaliana*.

**Electronic supplementary material:**

The online version of this article (doi:10.1186/s12870-015-0525-2) contains supplementary material, which is available to authorized users.

## Background

The endoplasmic reticulum (ER) hosts the synthesis and folding of proteins that are secreted extracellularly or delivered into different compartments of the endomembrane system. A significant portion of these proteins are *N*-glycosylated at an asparagine residue that is present in the consensus sequence -N-X-S/T- (where X is any amino acid except proline). This glycosylation occurs as they are translocated to the ER lumen by the reaction catalyzed by the enzyme oligosaccharyltransferase from a dolichol-PP-Glc3Man9GlcNAc2 oligosaccharide [1, 2]. Once the oligosaccharide is linked to asparagine, the last two glucoses are quickly removed by glucosidase I and glucosidase II to yield a protein with a bound GlcMan9GlcNAc2 oligosaccharide [3–5]. In addition, the nascent polypeptide begins to fold towards its proper conformation. This process is controlled by a mechanism known as the ER protein quality control (ER-QC) that includes the Calnexin (CNX)/Calreticulin (CRT) cycle.

The CNX and CRT are lectin/chaperones that bind the monoglucosylated oligosaccharides (GlcMan9GlcNAc2) present on *N*-glycosylated proteins they retain the proteins at the ER while they go through the folding process. Glucosidase II can cleave the remaining glucose residue to produce Man9GlcNAc2. If the protein is still not completely folded, it is recognized by the enzyme UDP-Glucose: Glycoprotein Glucosyltransferase (UGGT) that recognizes nearly folded proteins that lack glucose in *N*-oligosaccharide and catalyze the reglucosylation of these sugar moieties using UDP-glucose as substrate [6, 7]. Upon reglucosylation, the protein is again bound by CNX or CRT and retained in the ER to continue with the folding process. Glucosidase II then removes the Glc residue added by UGGT. Cycles of glucosylation-binding to CNX/CRT-deglucosylation continue until the glycoprotein folds or is targeted for degradation [8].

UGGT was described biochemically several years ago in many different species including plants [9]. Further studies helped to characterize the mechanism of action of the enzyme [10, 11]. However, little information is available regarding the physiological role that this enzyme plays. UGGT mutants in *S. pombe* show normal growth at standard conditions; however, the viability is reduced under extreme ER stress [12]. The absence of UGGT in mice results in embryo lethality suggesting a critical role of this enzyme in animals [13]. In Arabidopsis, Jin *et al.* [14] showed that a defective form of the brassinosteroid receptor (*bri1-9*) is retained in the ER but is released when the At1g71220 locus (encoding for the UGGT orthologue in Arabidopsis) was mutated in Arabidopsis.

Two other studies using forward genetic analysis concluded that this locus is also important for the biogenesis of the plant innate immune receptor EFR and suggested that EFR is a target of UGGT [15, 16]. Consequently, these results suggested that the At1g71220 locus is involved in ER-QC. However, in spite of the importance of these findings, no functional evidence on the actual activity of the gene product encoded by At1g71220 was provided. On the other hand, both Jin *et al.* [14] and Saijo *et al.* [16] indicated that— in contrast to what is observed in mice and despite the phenotypes observed at the molecular level— no obvious morphological phenotype was observed on mutants in the Arabidopsis UGGT orthologue. This suggested that this enzyme plays a less important role in plants than in animals.

To expand our understanding of the role that UGGT plays in the physiology of plants, we identified two allelic mutants on At1g71220 with abolished or significantly reduced UGGT activity in *Arabidopsis thaliana*. Both mutants showed a basal induction of the unfolded protein response (UPR) in the absence of any stimuli. Furthermore, they exhibited a delayed growth rate in the aerial part that became evident after 6 weeks of growth, even though after 10–12 weeks the wild type and the mutant plants showed no obvious morphological differences. Root growth was also affected in the mutants. Plants lacking UGGT showed a higher sensitivity to pathogen attack and a compromised basal and systemic resistance. These mutants are also more sensitive to heat, salt and salicylic acid during germination, which indicates that UGGT helps Arabidopsis cope with these stresses. Our results indicate that although mutations in UGGT are not lethal to *Arabidopsis thaliana,* this enzyme does play a significant role in plant growth and response to environmental cues.

## Results

### *Arabidopsis thaliana* ER-enriched fractions exhibit UGGT activity

The UGGT activity has been measured in mung bean [9], and its role in protein quality control in the ER has been determined based on genetic analyses in *Arabidopsis thaliana* [14–16]. These studies proposed that locus At1g71220 encodes for UGGT; however, the activity of its gene product has not been directly assessed. To analyze whether this locus encodes for UGGT, we measured its activity in Arabidopsis wild type and mutant plants in the locus At1g71220. The UGGT senses the conformation of the glycoproteins and transfers a Glc residue from UDP-Glc to Man9GlcNAc2-bearing proteins only if they have not yet acquired their native folding.

**Fig. 1:**
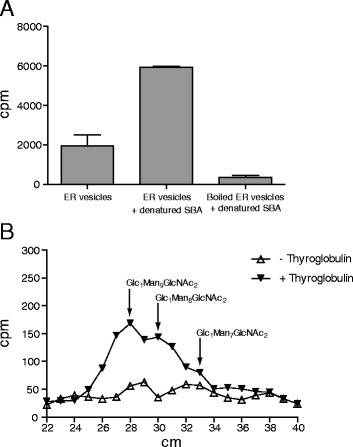
UGGT activity in ER-enriched fractions of *Arabidopsis thaliana.* **a** Microsomal membranes were prepared from etiolated Arabidopsis plants as described in the Material and Methods. The membranes were incubated in the presence of denatured SBA and UDP-[^14^C]glucose. The reaction was finished by adding TCA. The pellet was washed three times and the incorporated radioactivity was determined. Controls in which the SBA acceptor withheld or in which the ER-derived membranes were previously heat-inactivated are also shown. **b** The reaction was carried out as described above but with bovine thyroglobulin as the acceptor substrate. The proteins were treated with endoglycosidase H (Endo-H) to release the *N*-linked oligosaccharides that were separated by paper chromatography. The paper was cut and radioactivity determined by liquid scintillation

Using UDP-[^14^C]Glc and denatured soybean agglutinin (SBA, a glycoprotein that contains mainly Man9GlcNAc2 oligosaccharides) as substrates, we found that ER-enriched fractions from *Arabidopsis thaliana* increased the incorporation of the radioactive label into TCA-insoluble material. This indicates that UGGT activity was present in the ER fraction (Fig. 1a). The activity was abolished when the ER-enriched fraction was inactivated by boiling. We used thyroglobulin as an acceptor to confirm that glucose was transferred from UDP-Glc to high mannose glycoproteins; thyroglobulin is a glycoprotein that contains high mannose oligosaccharides (Man_9-7_GlcNAc2). After the reaction, the sample was treated with endo-β-N-acetylglucosaminidase H (endo-H) to release the *N*-linked oligosaccharides, which were then separated via paper chromatography as described by Trombetta et al. [9]. A peak migrating as the GlcMan9GlcNAc2 standard was observed after the treatment (Fig. 1b). We also observed two peaks with higher mobility that likely corresponded to GlcMan8GlcNAc2 and GlcMan_7_GlcNAc_2_. These results confirm that UGGT activity is present in ER-enriched fractions from *Arabidopsis thaliana*.

### Mutants in the UGGT-coding gene show a decrease in the glucosyltransferase activity

The *Arabidopsis thaliana* genome contains one locus (At1g71220) whose gene product shows similarity to UGGTs described in other species (Additional file 1). The protein bears the conserved C-terminal domain that contains the glucosyltransferase activity present in all UGGTs as well as a poorly conserved large N-terminal domain (Additional file 2). Orthologs of this gene are also present in other plant species (Additional file 1). *A. thaliana* expression databases indicate that *AtUGGT* is expressed in different organs. This was confirmed by quantitative PCR in which the *AtUGGT*-coding mRNA was detected at similar levels in roots, stems, leaves and flowers (Additional file 3).

**Fig. 2:**
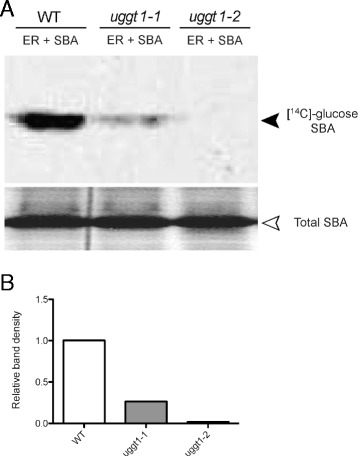
UGGT activity is reduced in plants bearing mutations in the At1g71220 gene. **a** UGGT activity detection in *A. thaliana* ER-enriched fractions from wild type and mutant plants. Incorporation of UDP-[^14^C]-glucose into unfolded SBA by wild type or mutant ER fractions. The upper panel shows the radioactivity associated with SBA while the lower panel shows the total amount of SBA used in the assay. **b** Quantification of the UGGT activity obtained from the PhosphorImager scans presented in **a**

To confirm that At1g71220 is indeed responsible for the UGGT activity detected on ER-enriched fractions, we analyzed whether mutants in this gene have diminished UGGT activity. Two insertional mutants were identified: *atuggt1-1* and *atuggt1-2* (Additional file 4). Homozygous plants were obtained for both alleles (Additional file 4). Gene expression analyses by quantitative PCR showed that both UGGT-coding mutant alleles have decreased mRNA transcript levels; however, these were not completely abolished (Additional file 4). To determine whether the mutants had less UGGT activity we incubated ER-enriched fractions with denatured SBA and UDP-[^14^C]glucose. After the reaction, proteins were separated on SDS-PAGE, and the radioactivity associated with SBA was assessed. The results indicated that *atuggt1-1* had some residual activity, whereas *atuggt1-2* had no detectable re-glucosylation activity (Fig. 2). These results strongly suggest that At1g71220 is responsible for the UGGT activity in Arabidopsis.

### AtUGGT expression is induced upon ER stress

**Fig. 3:**
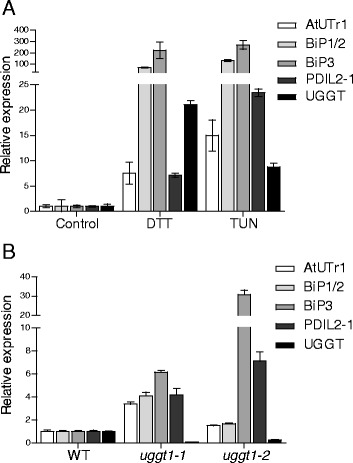
UGGT is induced during UPR and UGGT mutants that exhibit ER stress. **a** Quantitative real-time PCR monitoring of *AtUTr1, BiP1/2, BiP3, PDIL2-1* and *AtUGGT* transcript levels in stressed wild type plants. Fifteen day-old seedlings were treated over 5 hrs with DTT 2.5 mM or TUN 5 μg/ml in MS medium. Clathrin adapter (At5g46630) was used as a housekeeping gene. The average values of three independent experiments (*n* = 6) are shown; error bars represent ± SD. **b** Quantitative real-time PCR monitoring *AtUTr1, BiP1/2, BiP3, PDIL2-1* and *AtUGGT* transcript levels in wild type and AtUGGT mutant plants grown under normal conditions. Fifteen day-old seedlings were used for the analysis. Clathrin adapter (At5g46630) was the housekeeping gene. The average values of three independent experiments (*n* = 6) are shown; error bars represent ± SD

ER chaperones such as BiP are up-regulated by a signaling pathway known as UPR when ER stress is induced. Treatment of Arabidopsis plants with the ER stress-inducing agents tunicamycin or dithiothreitol (DTT) triggers UPR [17]. Because UGGT is a component of the CNX/CRT cycle [1], we wondered whether ER stress has any effect on the transcript levels of *AtUGGT*. Arabidopsis plants treated with tunicamycin and DTT showed an increased amount of *AtUGGT* at the transcript levels. This suggested that this gene is up-regulated by UPR. Other UPR-responding genes are involved in quality control and include *BIP1/2*, *BIP3* and *PDIL2-1* as well as *AtUTr1*, which is a gene encoding for an ER-localized UDP-glucose transporter likely involved in the supply of UDP-glucose for ER-QC [18]. These were also up-regulated under these conditions (Fig. 3a).

### UGGT mutants trigger UPR in the absence of an exogenous ER stress

We reasoned that a decrease in the activity of UGGT may perturb the mechanisms of quality control because the At*UGGT* transcript levels are increased by UPR. This caused the expression of other UPR-responding genes to change even in the absence of an exogenous ER stressor. Therefore, we assessed the transcript levels of different ER chaperones both in the wild type and in the two *AtUGGT* mutant alleles. The results pointed out that *AtUGGT* mutants exhibit an endogenous up-regulation of genes involved in quality control (*BiP1/2*, *BiP3*, *PDIL2-1*) as well as the UDP-glucose transporter *AtUTr1* (Fig. 3b).

**Fig. 4:**
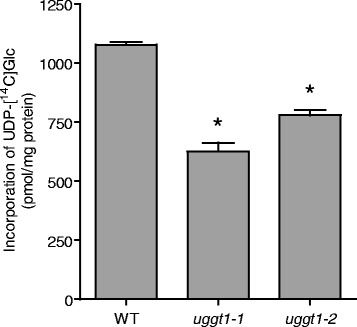
The *atuggt1-1* and *atuggt1-2* mutants exhibit a reduced uptake of UDP-[^14^C]Glc into ER-derived vesicles. The UDP-Glc uptake was assayed into 50 μg of ER-enriched fractions from wild-type and AtUGGT mutant plants incubated with 1 μM UDP-[^14^C]Glc for 15 min. The reaction was stopped with a 10-fold dilution with cold STM buffer and filtration. The filter-associated label was counted using liquid scintillation. Results are presented as mean with SD; significance was determined with ANOVA. Asterisks indicate a Tukey’s test p-value < 0.001

UDP-glucose is utilized by UGGT to re-glucosylate unfolded proteins within the ER. Our results showed that the UDP-glucose transporter gene *AtUTr1* is up-regulated upon ER-stress induction, but that it is also endogenously up-regulated in cells lacking UGGT. The up-regulation of the transporter suggests that the uptake of UDP-glucose could be enhanced in *AtUGGT* mutants. However, the lack of glucosyltransferase in the ER should reduce the usage of UDP-glucose in the ER and lead to a lower incorporation of UDP-glucose into this organelle. To address this issue, we assessed the incorporation of UDP-glucose into ER-enriched fractions from both wild type and *AtUGGT* mutant plants. Fig. 4 shows that although the nucleotide-sugar transporter coding-gene is up regulated in both UGGT mutant alleles, these plants show a decrease in the incorporation of UDP-Glc into ER fractions. This indicates that an active UGGT is important to drive the uptake of its substrate.

### Mutants in the *AtUGGT* gene exhibit an altered growth

**Fig. 5:**
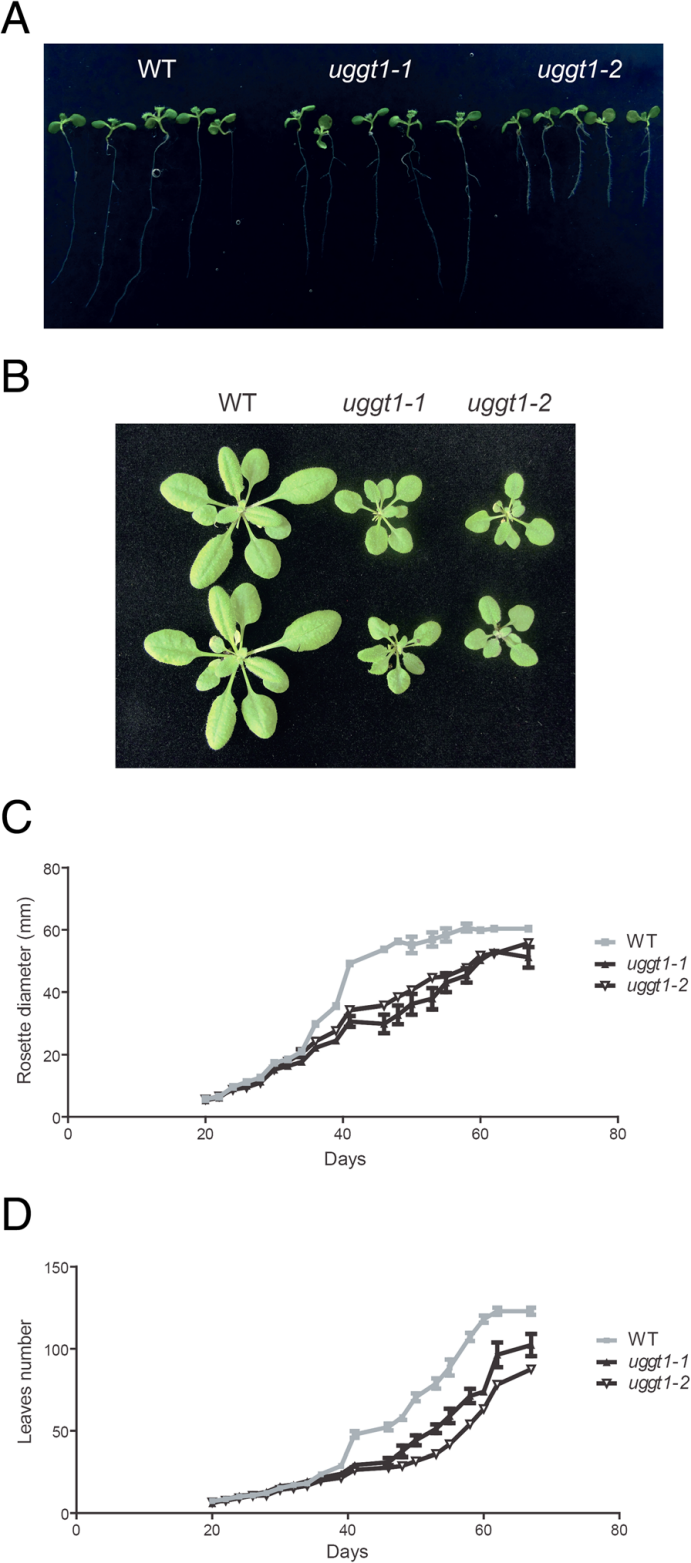
UGGT mutant plants show altered growth rates during vegetative development compared to wild type. **a** Root length in seven day-old seedlings grown in half MS medium; both wild type and AtUGGT mutant plants are shown. **b** Phenotypes of six-week-old plants grown in hydroponic medium. The rosette diameter (**c**) and the number of leaves (**d**) were measured in plants between days 20 and 70. The average values of eight independent plants (*n* = 8) are shown; error bars represent ± SD

Mutants in *UGGT* are lethal in mice [13]. On the other hand, *UGGT* mutants in *S. pombe* have no obvious phenotype in normal growth conditions although show a lethal phenotype upon ER stress induction [12]. Arabidopsis mutants in *AtUGGT* showed shorter roots when compared to wild type plants (Fig. 5a and Additional file 5). An analysis of the aerial growth part revealed that mutants had normal rosettes for about 35–40 days. After that time the mutants exhibited a delay in their growth rate and the number of leaves and size of the rosette were smaller (Fig. 5b, c, d). However, the differences were less evident after 60 days.

### *AtUGGT* mutant plants are more sensitive to biotic and abiotic stresses

**Fig. 6:**
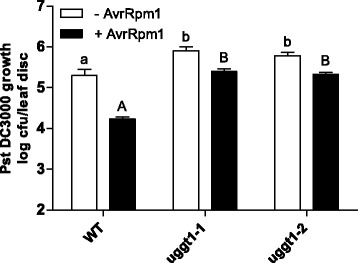
AtUGGT mutant plants are less tolerant to biotic stress. Whole leaves of four-week-old soil grown WT and mutant plants were infiltrated with *Pst* AvrRpm1 (OD600 = 0.001) to trigger SAR; a solution of 10 mM MgCl_2_ served as the mock. Twenty-four hours later the systemic leaves were infiltrated with *Pst* DC3000 (OD600 = 0.001). Bacterial growth (*Pst* DC3000) was monitored 3 days post infection. Error bars represent standard deviation from 6 samples. Different letters statically represent differences between the genotypes (lowercase for –AvrRpm1; uppercase for + AvrRpm1) at *p* < 0.05 (Tukey’s test). The experiments were performed at least three times with similar results

Multiple lines of evidence suggest that UGGT is involved in pathogen response [15, 16]. Therefore, we decided to test the sensitivity to pathogens on mutants that have residual or no detectable UGGT activity. We infected UGGT mutant plants with *Pseudomona syringae* pv tomato DC3000 (*Pst*) and assessed the number of bacteria infecting the leaves after 3 days. A higher number of bacteria were recovered from the leaves of mutants in comparison to those obtained from wild type suggesting that mutants in *AtUGGT* have an altered basal defense response (Fig. 6). Furthermore, a similar phenotype was observed when plants were first infected with *Pst* avrRpm1 to induce the systemic acquired resistance followed by infection with the virulent strain (Pst DC3000) (Fig. 6). These results indicate that both basal and systemic resistance responses are compromised in the *AtUGGT* mutants.

**Fig. 7:**
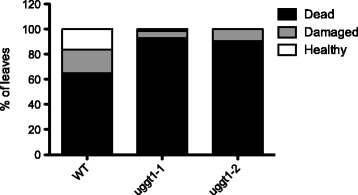
AtUGGT mutant plants are less tolerant to heat shock stress. Arabidopsis wild type and UGGT mutant plants were grown on soil for six weeks. The plants were treated at 42 °C for 2 hrs and returned to the growth chamber for 24 hrs. The leaves were then analyzed and classified as “dead” (completely dry and collapsed leaves), “damaged” (chlorotic lesions in leaves) or “healthy” (green and turgid leaves) and counted. The results are expressed as a percentage of total leaves analyzed per genotype (around 60 leaves per genotype)

**Fig. 8:**
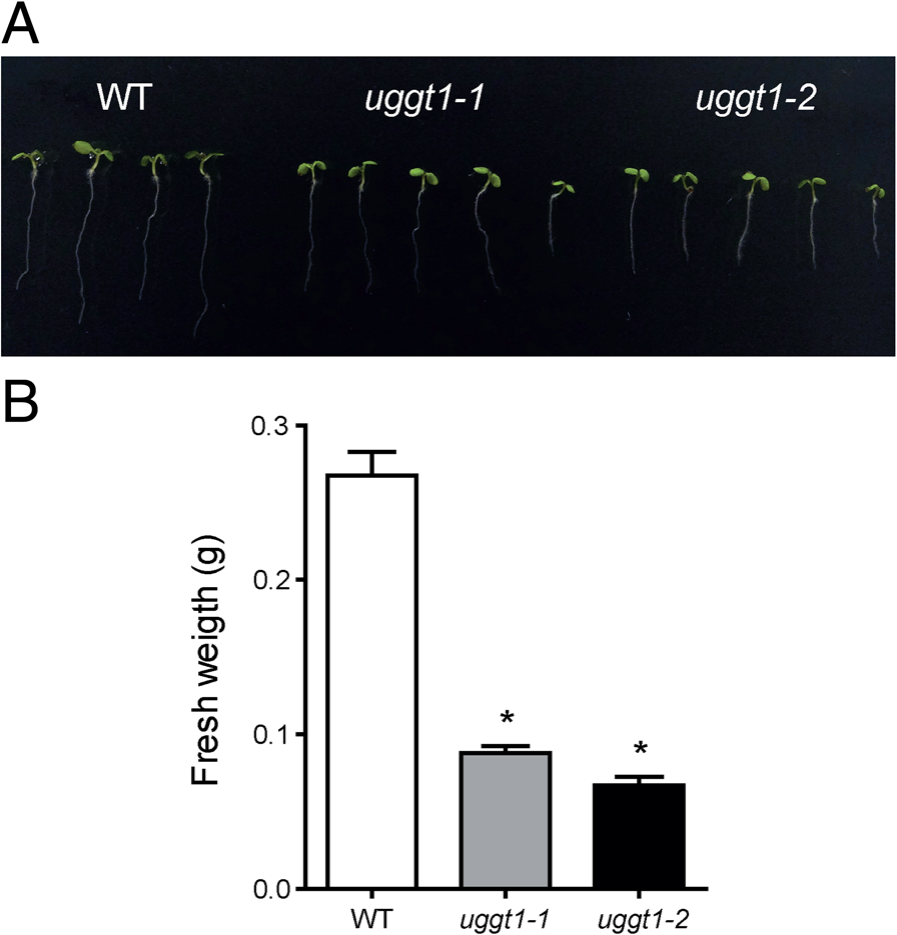
AtUGGT mutant plants are less tolerant to salt stress. **a** Photograph of seven-day-old seedlings of the different genotypes grown in MS media supplemented with 150 mM NaCl. **b** Arabidopsis wild type or UGGT mutants were grown in MS media supplemented with 150 mM NaCl for 2 weeks. Eighty plants of each genotype were weighed, and the experiments were performed in triplicate. The average values of three independent plates (*n* = 240) are shown; error bars represent ± SD. Statistical significance was determined by ANOVA. Asterisks indicate a Tukey's test p-value <0.01

We also investigated whether mutants in *AtUGGT* are more sensitive to abiotic stresses such as heat and salt because they have been shown to up-regulate ER chaperones associated with ERQC; mutants of the ERQC components also show a salt-sensitive phenotype [19–22]. Fig. 7 shows that mutant plants heat-shocked for 2 hrs at 42 °C and then returned to normal temperature developed a higher percentage of dead leaves and more chlorotic and necrotic lesions than wild type plants (Additional file 6). Furthermore, when grown at 150 mM NaCl, mutants were more sensitive than wild type plants (Fig. 8a) and displayed a significant decrease in fresh weight (Fig. 8b).

### Mutants in *AtUGGT* are over-sensitive to ER stress

**Fig. 9:**
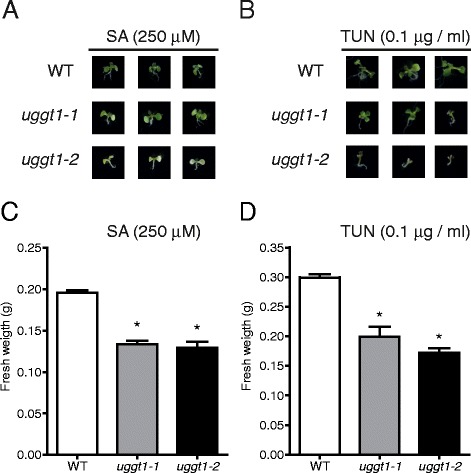
Mutant plants are over-sensitive to ER stress. Arabidopsis wild type and AtUGGT mutant plants were grown in MS media supplemented with 250 μM SA (**a** and **c**) or 0.1 μg/ml TUN (**b**) and (**d**) for 2 weeks. **a** and **b**. Photos of representative plants from different genotypes grown in SA or TUN, respectively. **c** and **d**. Eighty plants of each genotype were grown in SA or TUN, respectively, and were weighed in triplicates. The average values of three independent replicates (*n* = 240) are shown; error bars represent ± SD. Statistical significance was determined by ANOVA. Asterisks indicate a Tukey's test p-value < 0.01

To evaluate the sensitivity of the *AtUGGT* mutants to ER-stress, we grew the plants in the presence of tunicamycin and salicylic acid, which are two plant UPR-inducers [23]. Both allelic *AtUGGT* mutants were more sensitive to these compounds than the wild type (Fig. 9a and b). We also observed a significant decrease in fresh weight (Fig. 9c and d) when mutants were grown under these conditions. No differences were observed in the fresh weight of *AtUGGT* mutants compared to wild type plants in absence of ER stress (Additional file 7).

## Discussion and conclusion

UGGT is an enzyme that plays a critical role in the CNX/CRT cycle by sensing unfolded proteins and adding glucose to the *N*-linked oligosaccharide to form Glc1Man9GlcNAc2. CNX or CRT binds glycoproteins containing this oligosaccharide, which facilitate protein folding and enable interactions with ERp57 (a protein disulfide isomerase) [24]. This prevents aggregation of folding intermediates and maintains the glycoproteins at the ER until they are properly folded. This activity has been identified in different species ranging from protozoan to mammals [9]. Phylogenetic analyses show that different species share *UGGT* orthologs and that these are widely distributed in plants as well.

Our results show that UGGT is present in *Arabidopsis thaliana* and that there is a single locus (At1g71220) that accounts for this activity. Two allelic mutants in *AtUGGT* showed reduced or non-detectable transcript levels. Residual expression of genes is a phenomenon that has been observed in insertional mutants in different Arabidopsis loci [25]. Furthermore, while some low residual UGGT activity was observed in one of the alleles, we could not detect any activity in the second allele. All of this evidence strongly suggests that locus At1g71220 is responsible for the UGGT activity in Arabidopsis. In addition, it is likely that this is the only gene encoding for UGGT in *Arabidopsis thaliana* because no other homologous sequence is present in the genome.

Abolishing the expression of UGGT has different impacts on the viability of different species. *S. pombe* mutants in this gene are viable under normal conditions. However, their viability is affected under conditions of extreme ER stress [12]. On the other hand, the deletion of the UGGT gene in mice leads to embryo lethality although embryonic fibroblasts can propagate normally [13]. This suggests that in animals UGGT plays a fundamental role in the biogenesis and further localization of proteins that are involved in signaling among cells during the formation of multicellular structures. Data regarding the Arabidopsis UGGT mutants showed that they were viable with a normal reproductive cycle; however, different phenotypes were observed. At the macroscopic level, mutant plants were smaller than the wild type at some growth stages but eventually they reached similar sizes. Differences were more obvious in roots and rosette leaves.

The expression analyses of target genes that respond to the unfolding protein response showed that these genes were constitutively up-regulated in *AtUGGT* mutants. This indicates that decreasing the UGGT activity leads to ER stress. In particular, we observed that mutants in *AtUGGT* showed AtUTr1 up-regulation. This gene encodes for an ER-localized UDP-glucose transporter [26] that likely provides the substrate for UGGT. Nevertheless, the incorporation of UDP-glucose into ER-enriched vesicles is reduced in mutants in comparison to the wild type. This result suggests that UGGT is a driving force for the transport of UDP-glucose into the ER and that a deficiency in this enzyme leads to a reduction in the uptake of its substrate (Additional file 8). Interestingly, the incorporation of UDP-glucose into the ER was not completely reduced in mutants in UGGT suggesting that UDP-glucose is not only needed in the ER for protein re-glucosylation but by some other processes as well.

The decrease in UGGT activity has consequences for the response of these mutants to different stress conditions. We observed that *AtUGGT* mutants were more susceptible to pathogen infection at the basal and systemic level. This observation correlates with other reports that demonstrated an important role for the *AtUGGT* gene in the establishment of defense responses [15, 16].

In addition, growing these mutants in salicylic acid (SA) decreases their viability. Similar results were seen when plants are grown in tunicamycin—a chemical known to trigger the unfolded protein response (UPR). No differences in the fresh weight were observed when wild type and mutant plants were grown in the absence of tunicamycin or SA. Only the presence of these chemicals causes trait differences. This suggests that the endogenous ER stress level observed on AtUGGT mutants do not significantly alter the plant development at early developmental stages. A similar observation was described for the yeast UGGT mutant where cells are viable until an external cue that triggers the UPR is applied. This leads to cell death [12].

Interestingly, SA treatment also activates the UPR [23, 27] suggesting that an increase in the protein folding and secretion requires UGGT activity during the systemic acquire resistance. Furthermore, *AtUGGT* mutant plants also showed an increased susceptibility to several abiotic stress conditions. Indeed, leaves of these plants show a diminished percentage of survival under heat stress. Also these develop several chlorotic and necrotic lesions. In addition, the development of these mutants is severely affected by salt stress when compared to wild type plants.

Previous work indicated that Arabidopsis mutants in UGGT exhibit a phenotype at the molecular level regarding expression of genes involved in ERQC and the response to pathogens. However, no obvious growth phenotypes are seen when plants are grown normally without any treatment [14, 16], but no detailed phenotypic analyses of these mutants were done.

We performed an exhaustive analysis throughout the plant growth and under different growth conditions. The results showed no differences in the aerial part of the plant during the first 6 weeks of development. After that time however, we observed a slower growth rate in the mutants that led to smaller plants. The impaired growth was recovered after 10–12 weeks when wild type and mutants showed no differences. Although it could be argued that this phenomenon is a consequence of the growth conditions employed (hydroponic media), no studies have yet demonstrated that this condition triggers the UPR. It is difficult to predict what proteins are responsible for these phenotypes because UGGT is responsible for the proper folding of a number of proteins that are synthesized in the ER. Membrane receptors have been shown to depend on UGGT for their normal biosynthesis [15, 16] but it is also possible that other proteins involved in signaling are also altered in the mutant leading to a delayed growth rate.

The work of Liu et al. [28] supports this hypothesis. They showed that the expression in Arabidopsis of a constitutively active form of bZIP28, a transcription factor involved in the activation of UPR, produces a delayed growth of seedlings but with competent mature plants. This phenotype resembles in part the one observed in the *AtUGGT* mutant and suggests that continuous activation of UPR may delay the growth rate. Future work will further study this process.

Because heat stress perturbs the protein-folding processes, it is expected that mutants in the protein folding machinery display an altered response to heat stress. The *AtUGGT* mutants show extensive lesions in the aerial tissues of plant exposed to heat stress. Different reports show that heat stress induces UPR in *Arabidopsis thaliana* [22, 29]. These chlorotic and necrotic phenotypes can be associated to an overactive UPR. Also it is possible that these plants die because *AtUGGT* mutant plants display a constitutively activated UPR. In mammalian cells, the chronic activation of the UPR leads to cell death by apoptosis [30].

Regarding salt stress in the *AtUGGT* mutants, it is not clear how the CNX/CRT cycle and the activity of UGGT are related to this process. However, there is evidence of a relationship between salt stress and ER-QC/UPR. Li et al. [20], showed that mutants in CRT3 are more sensitive to salt during germination—this resembles the phenotype observed in the UGGT mutants. Therefore, it is likely that ER-QC and UPR are important in the plant response to salt stress.

Our results suggest that plants with decreased UGGT activity have an abnormal growth rate and are less tolerant to stress. This is in agreement with an increasing amount of evidence that supports the role of ER-QC and UPR in the plant response to different types of stresses [31, 32]. Because UGGT is a key component in the CNX/CRT cycle and ER-QC, our results provide additional support for the role of ER-QC in the plant response to environmental cues.

## Methods

### Plant material and treatments

The *Arabidopsis thaliana* wild type and UGGT mutants *uggt1-1 (CS854661)* and *uggt1-2 (SALK_016805)* are of Columbia (Col-0) background. For real time PCR analysis, seeds were germinated and grown *in vitro* in Murashige-Skoog (MS) medium supplemented with 15 g/l sucrose under controlled conditions in a growth chamber (16 hrs light, 100 μmoles m^−2^ s^−1^, 22 ± 2 °C). For treatments with tunicamycin (TUN) or ditiotreitol (DTT), 15 day-old seedlings were used. They were taken from the MS medium and placed in a Petri dish containing MS medium supplemented with 5 μg/ml TUN or 2.5 mM DTT for 5 hrs. Control samples were similarly incubated.

For hydroponic growing, seeds were germinated in hydroponic medium and grown under controlled conditions to plant senescence (approximately 70 days). For the plant fresh weight analysis under different chemical treatments, approximately 80 seeds were placed in petri dishes in triplicate. The seeds were germinated in MS solid medium supplemented with 0.1 μg/ml TUN, 250 μM SA or 150 mM NaCl for 10 days.

### Infection assays

*Pseudomonas* infection assays were performed as described previously [23]. Briefly, *Pseudomonas syringae* pv tomato DC3000 (*Pst*DC3000) and *Pst* avrRpm1 were grown at 28 °C on King’s B agar plates supplemented with 50 mg/ml rifampicin and 50 mg/ml kanamycin. Bacteria were suspended in 10 mM MgCl_2_ at OD_600_ = 0.001 and infiltrated into 3–4 leaves per plant leaf using a needleless syringe. Leaf discs from four independent plants were combined, ground in 10 mM MgCl_2_, serial-diluted 1:10 and plated onto King’s B medium containing the appropriate antibiotics. Plates were incubated at 28 °C for 3 days after which the colonies were counted.

To test for SAR, plants were pre-inoculated with *Pst* avrRpm1 (OD_600_ = 0.001) or mock (10 mM MgCl_2_) 24 hrs prior to infection. They were subsequently inoculated with *Pst*DC3000 (OD_600_ = 0.001) into 3–4 distal leaves per plant with 4 plants/genotype. Sampling was performed 3 days post inoculation.

### Phenotypic analysis of *A. thaliana* transgenic seedlings under stress conditions

Three replicates of 80 T3- mutant or wild-type seeds were placed in petri dishes containing MS medium supplemented with 150 mM NaCl or 250 μM SA. The plates were then transferred to the chamber, and seeds were germinated at 22 ± 2 °C under 16 hrs light/8 hrs dark at an illumination intensity of 100 μmol m^−2^ s^−1^ for 12 days.

To test for salt stress, seedlings were treated with liquid MS medium containing 150 mM NaCl for the defined times. For heat shock analysis, the mutant or wild-type plants were grown in soil for six weeks and then incubated in a chamber at 42 °C for 2 hrs. Finally, the plants were placed back in the growth chamber to observe recovery. The leaf phenotypes were analyzed after 24 hrs of the heat shock. Leaves were classified as “dead” (completely dry and collapsed leaves), “damaged” (chlorotic lesions in leaves) or “healthy” (green and turgid leaves). The results were graphed as the percentage of total leaves analyzed per genotype (around 60 leaves per genotype).

### Preparation of plant ER-enriched vesicles

Etiolated plants (50 g FW) were homogenized in 0.5 M sucrose and then were filtrated in miracloth. The filtrated material was centrifuged at 1000 × g for 2 min at 4 °C. The supernatant was layered over a 8 ml cushion of 1.3 M sucrose and then centrifuged at 100,000 × g for 90 min at 4 °C using a Sorvall AH-629 swinging bucket rotor. The upper phase was discarded leaving the membranous interphase. Layers of 1.1 M sucrose (15 ml) and 0.25 sucrose (5 ml) were added to the surface. This was centrifuged at 100,000 × g for 100 min at 4 °C. The membranous interphase between 1.3 and 1.1 M sucrose was withdrawn, and one volume of water was added followed by centrifugation at 100,000 × g for 50 min at 4 °C. The resulting pellet was resuspended in 500 μl of STM buffer (0.25 M sucrose, 10 mM pH 8 Tris-HCl and 1 mM MgCl2). A 20-μl aliquot was used for total protein quantification.

### UGGT activity

A 50-μl mixture containing 1 mg/ml of total proteins obtained from ER-enriched vesicles, 1.2 mg/ml of SBA, 200 μCi UDP-[^14^C]Glc, 10 mM HEPES, 20 mm CaCl_2_, 1.5 % Triton X-100 and 0.5 mM DNJ (chaperone 1-deoxynojirimycin) was incubated at 37 °C for 30 min.

The reaction was stopped by adding SDS-PAGE loading buffer and heating the mixture to 100 °C for 5 min. The samples were resolved by SDS-PAGE. The gel was dried-out overnight and the radioactive signals were quantified using a Phosphorimager (FX Molecular, BioRad).

### Uptake of UDP-[^14^C]glucose into ER vesicles

Sterile seeds of the wild type as well as *uggt1-1* and *uggt1-2* mutants were grown in a 16 hrs light/8 hrs dark cycle at 22 °C in MS containing 1 % sucrose (w/v) for 14 days. The plants were homogenized and subjected to subcellular fractionation as described by Muñoz et al. [33]. Endoplasmic reticulum-enriched microsomal fractions were taken from the 1.3/1.1 M sucrose interfaces as described above.

For the uptake assays, 50 μg of protein corresponding to the ER vesicles from the wild type, *uggt1-1* or *uggt1-2* plants were incubated with 1 μM UDP-[^14^C] glucose (0.1 μCi) in a medium containing 0.25 M sucrose, 10 mM Tris-HCl, pH 7.5 and 1 mM MgCl_2_ (STM buffer) for 15 min at 25 °C. To stop the reaction, the vesicles were diluted in cold STM buffer and filtered through 0.7-μm glass fiber filters. The filters were washed with an additional 10 volumes of cold STM buffer and dried. The radioactivity on the filters was determined by liquid scintillation counting.

### Quantitative PCR

Frozen plants were homogenized in liquid nitrogen using a mortar and pestle. Total RNA was isolated using Trizol® (Invitrogen, Karlsruhe, Germany), and residual DNA was removed with an RNase-free DNase I (Invitrogen, USA). One microgram total RNA was reverse transcribed using 500 ng of Oligo (dT) and 50 units of SuperScript II (Invitrogen, USA) following the supplier’s instructions. Quantitative real time PCR was performed using the Fast Eva Green Master mix (Biotium, USA). The PCR conditions consisted of 40 cycles of denaturation at 95 °C for 15 s, annealing at 55 or 60 °C for 15 s and an extension at 72 °C for 15 s. A dissociation curve was generated at the end of each PCR cycle to verify that a single product was amplified using the software provided with the Stratagene System.

To minimize sample variations, mRNA expression of the target gene was normalized relative to the expression of the clathrin adaptor housekeeping gene. The experiments were repeated four times. Quantification of clathrin adaptor (At5G46630) mRNA levels in the threshold cycle (Ct) internal standard was subtracted from values from genes of interest to obtain a ΔCt value. The Ct value of untreated control sample was subtracted from the ΔCt value to obtain a ΔΔCt value. The fold changes in expression level relative to the control were expressed as a 2-ΔΔCt. The following primers were designed for gene-specific transcript amplification:

*UGGT (*AT1G71220); UGGT-*F*: GGGACCACCACCAATCTG, UGGT-*R*: CCATCGGAACCAAGCCAAG; *AtUTr1* (AT2G02810); AtUTr1-*F*: AAAAGAGTTGAAGTTTTTCCC, AtUTr1-*R*: ATCCACAAAATTCAAATCATATAT; *BiP 1/2* (AT5G28540 and AT5G42020); BiP1/2-*F*: ATATGGCTCGCTCGTTTGG, BiP1/2-*R*: GGTTTCCTTGGTCATTGGCA; BiP3 (AT1G09080); BiP3-*F*: CACGGTTCCAGCGTATTTCAAT, BiP3-R: ATAAGCTATGGCAGCACCCGTT; *PDIL1-2* (AT1g77510); PDIL2-1-*F*: CACACAAAGCCCTTGGCGAGAAAT, PDIL2-1-*R*: AATCCCTGCCACCGTCATAATCGT, clathrin adaptor (AT5G46630); CLAT-*F*: GAAACATGGTGGATGCAT; and CLAT-*R*: CTCAACACCAAATTTGAATC.

## Additional files

Additional file 1:
***Arabidopsis thaliana*** **gene At1g71220 is an ortholog of eukaryotic UGGTs.** A) Phylogenetic tree comprising most of the described UGGTs. Amino acid sequences were retrieved from NCBI Homologene Database (http://www.ncbi.nlm.nih.gov/homologene/) aligned using Clustal Omega (Sievers et al., 2011). A phylogenetic tree was generated using MEGA 5 (Tamura et al., 2011). The neighbor-joining method and a bootstrap calculation with 5000 iterations were used for tree generation. Accession numbers for each sequence are: *Schizosaccharomyces pombe* (NP_595281.1); *Magnaporthe oryzae* (XP_360967.2); *Neurospora crassa* (XP_959471.1); *Arabidopsis thaliana* (NP_177278.3); *Drosophila melanogaster* (NP_524151.2); *Anopheles gambiae* (XP_313307.4); *Rattus novergicus* (NP_598280.1); *Caenorhabditis elegans*_UGGT1 (NP_509268.1); *Caenorhabditis elegans*_UGGT2 (NP_492484.2); *Danio rerio*_UGGT1 (NP_001071002.1); *Danio rerio*_UGGT2 (XP_697781.2); *Gallus gallus*_UGGT1 (XP_422579.3); *Gallus gallus*_UGGT2 (NP_001239028.1); *Mus musculus*_UGGT1 (NP_942602.2); *Mus musculus*_UGGT2 (NP_001074721.2); *Bos taurus*_UGGT1 (XP_002685277.1); *Bos taurus*_UGGT2 (XP_002692017.1); *Canis lupus*_UGGT1 (XP_533310.3); *Canis lupus*_UGGT2 (XP_542644.3); *Macaca mulatta*_UGGT1 (XP_001091373.1); *Macaca mulatta*_UGGT2 (XP_001086327.2); *Pan troglodytes*_UGGT1 (XP_001141314.1); *Pan troglodytes*_UGGT2 (XP_001139906.1); *Homo sapiens*_UGGT1 (NP_064505.1); and *Homo sapiens*_UGGT2 (NP_064506.3). B) Phylogenetic tree of the UGGTs present in plants and green algae. Amino acid sequences were retrieved from Phytozome Database (http://phytozome.jgi.doe.gov/pz/portal.html) and aligned using Clustal Omega [34]. A phylogenetic tree was generated using MEGA 5 [35]. The neighbor-joining method and a bootstrap calculation with 5000 iterations were used for tree generation. Both trees were visualized using Fig Tree v1.4.0 (http://tree.bio.ed.ac.uk/software/figtree/) to display bootstrap values. Additional file 2:
**The C-terminal sequence of Arabidopsis UGGT is highly conserved among eukaryotes.** Several eukaryotic UGGT sequences were retrieved using the NCBI Homologene database (http://www.ncbi.nlm.nih.gov/homologene/). These were aligned using Clustal Omega [34] and visualized with Jalview [36]. The highly conserved region near the C-terminal part of all UGGTs is shown. Accession numbers for each sequence are: *Schizosaccharomyces pombe* (NP_595281.1); *Magnaporthe oryzae* (XP_360967.2); *Neurospora crassa* (XP_959471.1); *Arabidopsis thaliana* (NP_177278.3); *Drosophila melanogaster* (NP_524151.2); *Anopheles gambiae* (XP_313307.4); *Rattus novergicus* (NP_598280.1); *Caenorhabditis elegans*_UGGT1 (NP_509268.1); *Caenorhabditis elegans*_UGGT2 (NP_492484.2); *Danio rerio*_UGGT1 (NP_001071002.1); *Danio rerio*_UGGT2 (XP_697781.2); *Gallus gallus*_UGGT1 (XP_422579.3); *Gallus gallus*_UGGT2 (NP_001239028.1); *Mus musculus*_UGGT1 (NP_942602.2); *Mus musculus*_UGGT2 (NP_001074721.2); *Bos taurus*_UGGT1 (XP_002685277.1); *Bos taurus*_UGGT2 (XP_002692017.1); *Canis lupus*_UGGT1 (XP_533310.3); *Canis lupus*_UGGT2 (XP_542644.3); *Macaca mulatta*_UGGT1 (XP_001091373.1); *Macaca mulatta*_UGGT2 (XP_001086327.2); *Pan troglodytes*_UGGT1(XP_001141314.1); *Pan troglodytes*_UGGT2 (XP_001139906.1); *Homo sapiens*_UGGT1 (NP_064505.1); and *Homo sapiens*_UGGT2 (NP_064506.3). Additional file 3:
**Arabidopsis UGGT mRNA is expressed in different tissues.** Quantitative real-time PCR monitoring of UGGT transcript levels in the indicated tissues was performed using six-week-old plants. Clathrin adapter (At5g46630) was used as a housekeeping gene to normalize expression values. The average values of three independent experiments (*n* = 6) are shown; error bars represent ± SD. Additional file 4:
**Identification of homozygous T-DNA insertional mutants on UGGT gene.** A) Schematic representation of UGGT gene structure. Boxes represent exons and lines introns. The T-DNA insertions are indicated as *uggt1-1* and *uggt1-2*. Black arrows indicate primers used to amplify wild type allele. Red arrows indicate primers for left border of T-DNA insertions used to amplify mutant alleles. B) Amplification of wild type or mutant allele of At1g71220 on different T-DNA insertional mutants. The primers were as follows: LBp745: AACGTCCGCAATGTGTTATTAAGTTGTC; UGGT1: CTAATGGCCTGTGTTCCTCTCA; UGGT2: GTCAGCAATGCCAGGAAAGTGC; LBb1.3: ATTTTGCCGATTTCGGAAC; UGGT5: 5CCTTTATTGTGGTTACTGGTAC; and UGGT8: CTGTACTGCTGTAATCGTCCT. a) Amplification of the wild type allele using primers UGGT1 and UGGT2. b) Amplification of the mutant allele in the *uggt1-1* genotype using primers LBDsLox and UGGT2. c) Amplification of the wild type allele using the primers UGGT5 and UGGT8. d) Amplification of mutant allele in the *uggt1-2* genotype using primers UGGT5 and LBb1.3. Amplicons separated in an agarose gel are shown. WT: wild type; *uggt1-1*: line CS854661; *uggt1-*2: line SALK_016805; −: control using water instead of template. C) Quantitative real-time PCR monitoring of UGGT transcript levels in the indicated genotypes. Clatrin adapter (At5g46630) was used as a control. The average values of three independent experiments (*n* = 6) are shown; error bars represent ± SD. Additional file 5:
**Arabidopsis UGGT mutants have shorter roots than wild type plants.** *Arabidopsis thaliana* plants were grown in hydroponic media for 3 weeks and then the roots were photographed. A representative plant of each genotype is shown. Additional file 6:
**Leaves of Arabidopsis UGGT mutants show extensive chlorotic lesions after heat treatment.** Arabidopsis plants grown on soil for 4 weeks were exposed to 42 °C for 2 hrs. The plants were recovered for 24 hrs and the leaves were photographed. Additional file 7:
**Arabidopsis UGGT mutant plants have no differences in fresh weight compared to wild type plants under non-ER stress conditions.** Arabidopsis wild type or UGGT mutants were grown in MS media for 2 weeks. Eighty plants of each genotype were weighed, and the experiments were performed in triplicate. The average values of three independent plates (*n* = 240) are shown; error bars represent ± SD. Additional file 8:
**Model of UDP-Glucose (UDP-Glc) incorporation and utilization by UGGT.** The scheme shows the wild type situation where UGGT located in the ER lumen transfer glucose from UDP-Glc to nearly folded proteins. UDP-Glc is incorporated from the cytosol into the lumen through the UDP-Glc transporters AtUTr1 and AtUTr3. These are antiporters, and they use UMP as exchanger. Thus, the UGGT activity drives a cycle that stimulates the incorporation of UDP-glucose. In contrast, the absence of UGGT in the mutant leads to a decrease in the uptake of UDP-Glc despite the increase in UDP-glucose transporters that is likely due to the lack of UMP. In addition, unfolded proteins accumulate and trigger the unfolded protein response (UPR). Finally, another pathway that may use UDP-glucose in the ER is the reaction catalyzed by the product of the putative ALG5 gene from Arabidopsis. This transfers glucose from UDP-glucose into dolichol—an ER-anchored lipid. This reaction may explain the residual signal observed in the UDP-glucose incorporation assays in ER vesicles isolated from AtUGGT mutants (Fig. 4).

## Abbreviations

ER: Endoplasmic reticulum
ER-QC: ER protein quality control
CNX: Calnexin
CRT: Calreticulin
UGGT: UDP-Glucose: Glycoprotein Glucosyltransferase
UPR: Unfolded protein response
SAR: Systemic acquired resistance
SBA: Soybean agglutinin
endo-H: endo-β-N-acetylglucosaminidase H
DTT: Dithiothreitol
TUN: Tunicamycin
*Pst*: *Pseudomona syringae* pv tomato DC3000
MS: Murashige-Skoog
