# Supplementary figures and images for: The UDP-glucose: glycoprotein glucosyltransferase (UGGT), a key enzyme in ER quality control, plays a significant role in plant growth as well as biotic and abiotic stress in Arabidopsis thaliana
Source: BMC Plant Biol. 2015 May 28;15:127. doi: 10.1186/s12870-015-0525-2 (PMC4465474; doi:10.1186/s12870-015-0525-2)

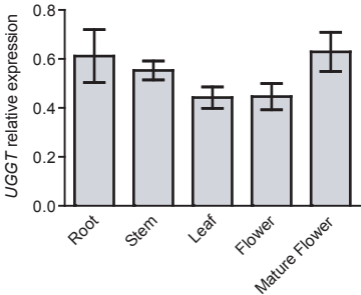

Supplement: Additional file 3: — Arabidopsis UGGT mRNA is expressed in different tissues. Quantitative real-time PCR monitoring of UGGT transcript levels in the indicated tissues was performed using six-week-old plants. Clathrin adapter (At5g46630) was used as a housekeeping gene to normalize expression values. The average values of three independent experiments (n = 6) are shown; error bars represent ± SD. [file 12870_2015_525_MOESM3_ESM.pdf]

A

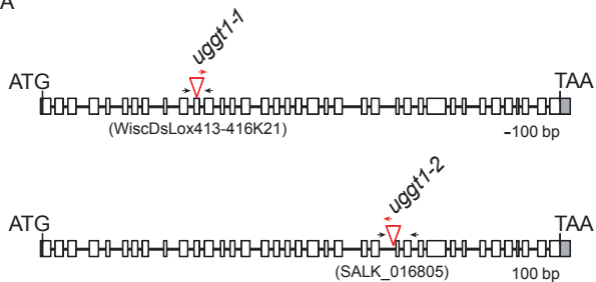

B

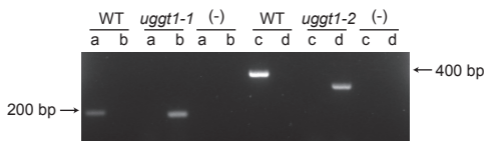

C

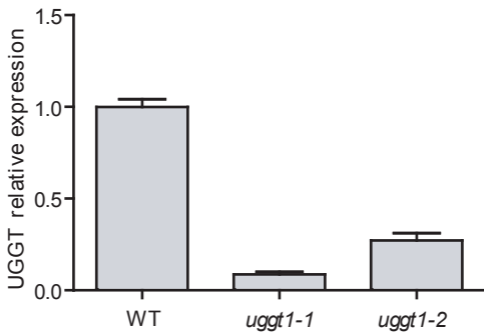

Supplement: Additional file 4: — Identification of homozygous T-DNA insertional mutants on UGGT gene. A) Schematic representation of UGGT gene structure. Boxes represent exons and lines introns. The T-DNA insertions are indicated as uggt1-1 and uggt1-2. Black arrows indicate primers used to amplify wild type allele. Red arrows indicate primers for left border of T-DNA insertions used to amplify mutant alleles. B) Amplification of wild type or mutant allele of At1g71220 on different T-DNA insertional mutants. The primers were as follows: LBp745: AACGTCCGCAATGTGTTATTAAGTTGTC; UGGT1: CTAATGGCCTGTGTTCCTCTCA; UGGT2: GTCAGCAATGCCAGGAAAGTGC; LBb1.3: ATTTTGCCGATTTCGGAAC; UGGT5: 5CCTTTATTGTGGTTACTGGTAC; and UGGT8: CTGTACTGCTGTAATCGTCCT. a) Amplification of the wild type allele using primers UGGT1 and UGGT2. b) Amplification of the mutant allele in the uggt1-1 genotype using primers LBDsLox and UGGT2. c) Amplification of the wild type allele using the primers UGGT5 and UGGT8. d) Amplification of mutant allele in the uggt1-2 genotype using primers UGGT5 and LBb1.3. Amplicons separated in an agarose gel are shown. WT: wild type; uggt1-1: line CS854661; uggt1-2: line SALK_016805; −: control using water instead of template. C) Quantitative real-time PCR monitoring of UGGT transcript levels in the indicated genotypes. Clatrin adapter (At5g46630) was used as a control. The average values of three independent experiments (n = 6) are shown; error bars represent ± SD. [file 12870_2015_525_MOESM4_ESM.pdf]

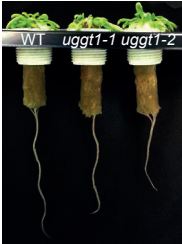

Supplement: Additional file 5: — Arabidopsis UGGT mutants have shorter roots than wild type plants. Arabidopsis thaliana plants were grown in hydroponic media for 3 weeks and then the roots were photographed. A representative plant of each genotype is shown. [file 12870_2015_525_MOESM5_ESM.pdf]

WT

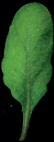

*uggt1-1*

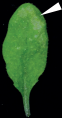

*uggt1-2*

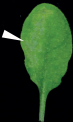

Supplement: Additional file 6: — Leaves of Arabidopsis UGGT mutants show extensive chlorotic lesions after heat treatment. Arabidopsis plants grown on soil for 4 weeks were exposed to 42 °C for 2 hrs. The plants were recovered for 24 hrs and the leaves were photographed. [file 12870_2015_525_MOESM6_ESM.pdf]

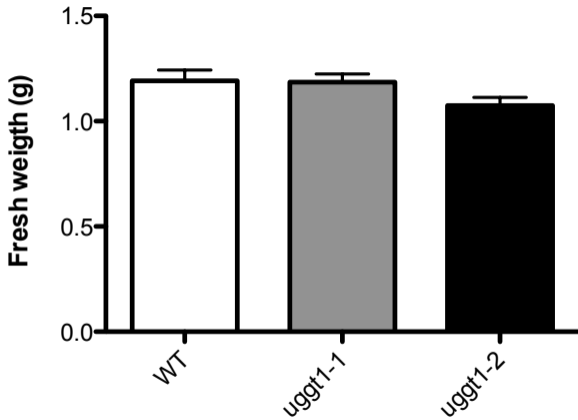

Supplement: Additional file 7: — Arabidopsis UGGT mutant plants have no differences in fresh weight compared to wild type plants under non-ER stress conditions. Arabidopsis wild type or UGGT mutants were grown in MS media for 2 weeks. Eighty plants of each genotype were weighed, and the experiments were performed in triplicate. The average values of three independent plates (n = 240) are shown; error bars represent ± SD. [file 12870_2015_525_MOESM7_ESM.pdf]

WT

*uggt* mutant

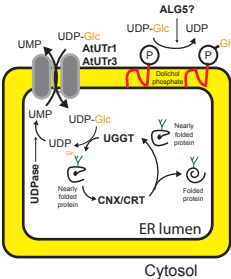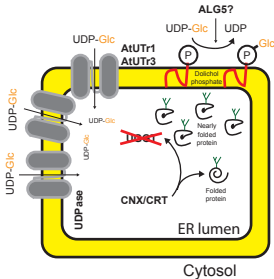

Supplement: Additional file 8: — Model of UDP-Glucose (UDP-Glc) incorporation and utilization by UGGT. The scheme shows the wild type situation where UGGT located in the ER lumen transfer glucose from UDP-Glc to nearly folded proteins. UDP-Glc is incorporated from the cytosol into the lumen through the UDP-Glc transporters AtUTr1 and AtUTr3. These are antiporters, and they use UMP as exchanger. Thus, the UGGT activity drives a cycle that stimulates the incorporation of UDP-glucose. In contrast, the absence of UGGT in the mutant leads to a decrease in the uptake of UDP-Glc despite the increase in UDP-glucose transporters that is likely due to the lack of UMP. In addition, unfolded proteins accumulate and trigger the unfolded protein response (UPR). Finally, another pathway that may use UDP-glucose in the ER is the reaction catalyzed by the product of the putative ALG5 gene from Arabidopsis. This transfers glucose from UDP-glucose into dolichol—an ER-anchored lipid. This reaction may explain the residual signal observed in the UDP-glucose incorporation assays in ER vesicles isolated from AtUGGT mutants (Fig. 4). [file 12870_2015_525_MOESM8_ESM.pdf]
