# Supplementary material for: The UDP-glucose: glycoprotein glucosyltransferase (UGGT), a key enzyme in ER quality control, plays a significant role in plant growth as well as biotic and abiotic stress in Arabidopsis thaliana
Source: BMC Plant Biol. 2015 May 28;15:127. doi: 10.1186/s12870-015-0525-2 (PMC4465474; doi:10.1186/s12870-015-0525-2)
Supplement: Additional file 1: — Arabidopsis thaliana gene At1g71220 is an ortholog of eukaryotic UGGTs. A) Phylogenetic tree comprising most of the described UGGTs. Amino acid sequences were retrieved from NCBI Homologene Database (http://www.ncbi.nlm.nih.gov/homologene/) aligned using Clustal Omega (Sievers et al., 2011). A phylogenetic tree was generated using MEGA 5 (Tamura et al., 2011). The neighbor-joining method and a bootstrap calculation with 5000 iterations were used for tree generation. Accession numbers for each sequence are: Schizosaccharomyces pombe (NP_595281.1); Magnaporthe oryzae (XP_360967.2); Neurospora crassa (XP_959471.1); Arabidopsis thaliana (NP_177278.3); Drosophila melanogaster (NP_524151.2); Anopheles gambiae (XP_313307.4); Rattus novergicus (NP_598280.1); Caenorhabditis elegans_UGGT1 (NP_509268.1); Caenorhabditis elegans_UGGT2 (NP_492484.2); Danio rerio_UGGT1 (NP_001071002.1); Danio rerio_UGGT2 (XP_697781.2); Gallus gallus_UGGT1 (XP_422579.3); Gallus gallus_UGGT2 (NP_001239028.1); Mus musculus_UGGT1 (NP_942602.2); Mus musculus_UGGT2 (NP_001074721.2); Bos taurus_UGGT1 (XP_002685277.1); Bos taurus_UGGT2 (XP_002692017.1); Canis lupus_UGGT1 (XP_533310.3); Canis lupus_UGGT2 (XP_542644.3); Macaca mulatta_UGGT1 (XP_001091373.1); Macaca mulatta_UGGT2 (XP_001086327.2); Pan troglodytes_UGGT1 (XP_001141314.1); Pan troglodytes_UGGT2 (XP_001139906.1); Homo sapiens_UGGT1 (NP_064505.1); and Homo sapiens_UGGT2 (NP_064506.3). B) Phylogenetic tree of the UGGTs present in plants and green algae. Amino acid sequences were retrieved from Phytozome Database (http://phytozome.jgi.doe.gov/pz/portal.html) and aligned using Clustal Omega [34]. A phylogenetic tree was generated using MEGA 5 [35]. The neighbor-joining method and a bootstrap calculation with 5000 iterations were used for tree generation. Both trees were visualized using Fig Tree v1.4.0 (http://tree.bio.ed.ac.uk/software/figtree/) to display bootstrap values. [file 12870_2015_525_MOESM1_ESM.pdf]

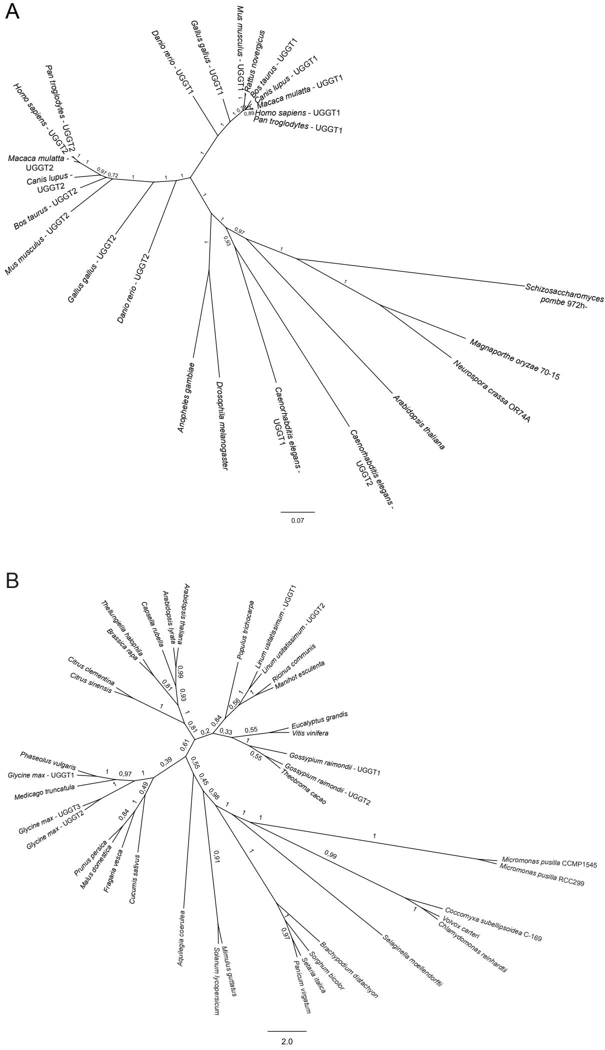
A
Mus musculus - UGGT1
Rattus novergicus
Gallus gallus - UGGT1
Danio rerio - UGGT1
Bos taurus - UGGT1
Canis lupus - UGGT1
Macaca mulatta - UGGT1
Homo sapiens - UGGT1
Pan troglodytes - UGGT1
Pan troglodytes - UGGT2
Homo sapiens - UGGT2
Macaca mulatta - UGGT2
Canis lupus - UGGT2
Bos taurus - UGGT2
Mus musculus - UGGT2
Gallus gallus - UGGT2
Danio rerio - UGGT2
Schizosaccharomyces pombe 972h-
Magnaporthe oryzae 70-15
Neurospora crassa OR74A
Arabidopsis thaliana
Caenorhabditis elegans - UGGT2
Caenorhabditis elegans - UGGT1
Drosophila melanogaster
Anopheles gambiae
0.07
B
Arabidopsis thaliana
Arabidopsis lyrata
Capsella rubella
Thellungiella halophila
Brassica rapa
Citrus clementina
Citrus sinensis
Populus trichocarpa
Linum usitatissimum - UGGT1
Linum usitatissimum - UGGT2
Ricinus communis
Manihot esculenta
Eucalyptus grandis
Vitis vinifera
Gossypium raimondii - UGGT1
Gossypium raimondii - UGGT2
Theobroma cacao
Phaseolus vulgaris
Glycine max - UGGT1
Medicago truncatula
Glycine max - UGGT3
Glycine max - UGGT2
Prunus persica
Malus domestica
Fragaria vesca
Cucumis sativus
Aquilegia coerulea
Mimulus guttatus
Solanum lycopersicum
Brachypodium distachyon
Sorghum bicolor
Setaria italica
Panicum virgatum
Selaginella moellendorffii
Coccomyxa subellipsoidea C-169
Volvox carteri
Chlamydomonas reinhardtii
Micromonas pusilla CCMP1545
Micromonas pusilla RCC299
2.0
