# Supplementary material for: The UDP-glucose: glycoprotein glucosyltransferase (UGGT), a key enzyme in ER quality control, plays a significant role in plant growth as well as biotic and abiotic stress in Arabidopsis thaliana
Source: BMC Plant Biol. 2015 May 28;15:127. doi: 10.1186/s12870-015-0525-2 (PMC4465474; doi:10.1186/s12870-015-0525-2)
Supplement: Additional file 2: — The C-terminal sequence of Arabidopsis UGGT is highly conserved among eukaryotes. Several eukaryotic UGGT sequences were retrieved using the NCBI Homologene database (http://www.ncbi.nlm.nih.gov/homologene/). These were aligned using Clustal Omega [34] and visualized with Jalview [36]. The highly conserved region near the C-terminal part of all UGGTs is shown. Accession numbers for each sequence are: Schizosaccharomyces pombe (NP_595281.1); Magnaporthe oryzae (XP_360967.2); Neurospora crassa (XP_959471.1); Arabidopsis thaliana (NP_177278.3); Drosophila melanogaster (NP_524151.2); Anopheles gambiae (XP_313307.4); Rattus novergicus (NP_598280.1); Caenorhabditis elegans_UGGT1 (NP_509268.1); Caenorhabditis elegans_UGGT2 (NP_492484.2); Danio rerio_UGGT1 (NP_001071002.1); Danio rerio_UGGT2 (XP_697781.2); Gallus gallus_UGGT1 (XP_422579.3); Gallus gallus_UGGT2 (NP_001239028.1); Mus musculus_UGGT1 (NP_942602.2); Mus musculus_UGGT2 (NP_001074721.2); Bos taurus_UGGT1 (XP_002685277.1); Bos taurus_UGGT2 (XP_002692017.1); Canis lupus_UGGT1 (XP_533310.3); Canis lupus_UGGT2 (XP_542644.3); Macaca mulatta_UGGT1 (XP_001091373.1); Macaca mulatta_UGGT2 (XP_001086327.2); Pan troglodytes_UGGT1(XP_001141314.1); Pan troglodytes_UGGT2 (XP_001139906.1); Homo sapiens_UGGT1 (NP_064505.1); and Homo sapiens_UGGT2 (NP_064506.3). [file 12870_2015_525_MOESM2_ESM.pdf]

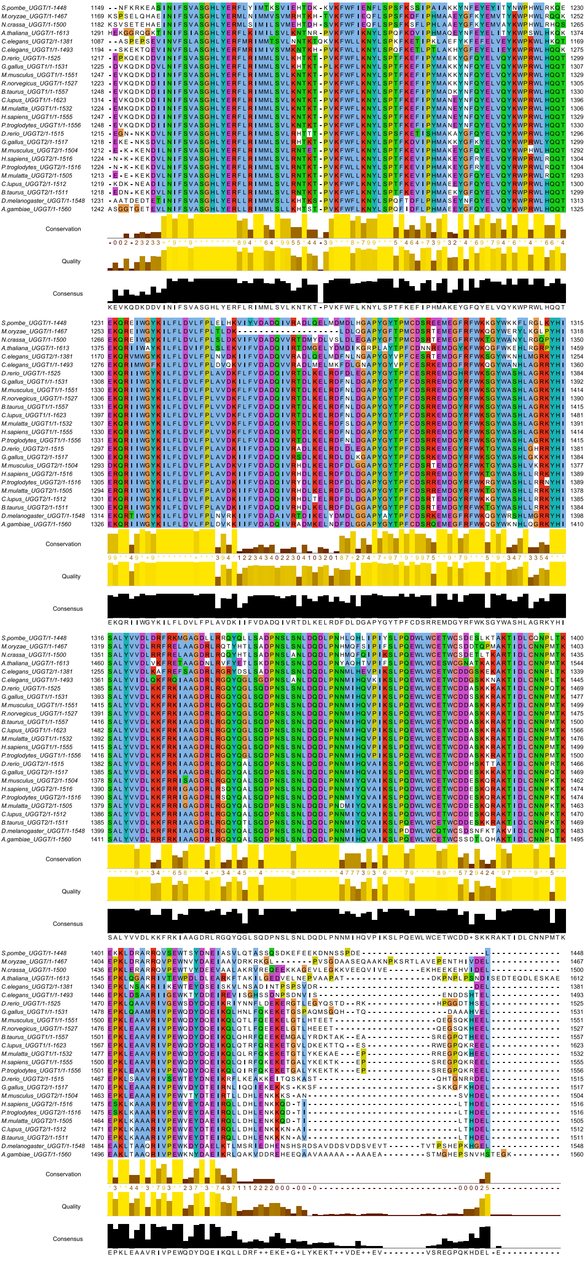
S.pombe_UGGT/1-1448
M.oryzae_UGGT/1-1467
N.crassa_UGGT/1-1500
A.thaliana_UGGT/1-1613
C.elegans_UGGT2/1-1381
C.elegans_UGGT1/1-1493
D.rerio_UGGT1/1-1525
G.gallus_UGGT1/1-1531
M.musculus_UGGT1/1-1551
R.norvegicus_UGGT1/1-1527
B.taurus_UGGT1/1-1557
C.lupus_UGGT1/1-1623
M.mulatta_UGGT1/1-1532
H.sapiens_UGGT1/1-1555
P.troglodytes_UGGT1/1-1556
D.rerio_UGGT2/1-1515
G.gallus_UGGT2/1-1517
M.musculus_UGGT2/1-1504
H.sapiens_UGGT2/1-1516
P.troglodytes_UGGT2/1-1516
M.mulatta_UGGT2/1-1505
C.lupus_UGGT2/1-1512
B.taurus_UGGT2/1-1511
D.melanogaster_UGGT/1-1548
A.gambiae_UGGT/1-1560
Conservation
Quality
Consensus
